# Functional sympatholysis and sympathetic escape in a theoretical model for blood flow regulation

**DOI:** 10.3389/fphys.2014.00192

**Published:** 2014-05-26

**Authors:** Tuhin K. Roy, Timothy W. Secomb

**Affiliations:** ^1^Department of Anesthesiology, Mayo ClinicRochester, MN, USA; ^2^Department of Physiology, Arizona Health Sciences Center, University of ArizonaTucson, AZ, USA

**Keywords:** sympathetic nerve activity, microvascular flow regulation, mathematical model, exercise

## Abstract

A mathematical simulation of flow regulation in vascular networks is used to investigate the interaction between arteriolar vasoconstriction due to sympathetic nerve activity (SNA) and vasodilation due to increased oxygen demand. A network with 13 vessel segments in series is used, each segment representing a different size range of arterioles or venules. The network includes five actively regulating arteriolar segments with time-dependent diameters influenced by shear stress, wall tension, metabolic regulation, and SNA. Metabolic signals are assumed to be propagated upstream along vessel walls via a conducted response. The model exhibits functional sympatholysis, in which sympathetic vasoconstriction is partially abrogated by increases in metabolic demand, and sympathetic escape, in which SNA elicits an initial vasoconstriction followed by vasodilation. In accordance with experimental observations, these phenomena are more prominent in small arterioles than in larger arterioles when SNA is assumed to act equally on arterioles of all sizes. The results imply that a mechanism based on the competing effects on arteriolar tone of SNA and conducted metabolic signals can account for several observed characteristics of functional sympatholysis, including the different responses of large and small arterioles.

## Introduction

Local regulation of blood flow is critical for coordinating perfusion with local metabolic demand. Even under conditions of adequate overall organ blood flow, impairment of local regulation can lead to areas of inadequate oxygen delivery and impaired oxygen extraction. During flow regulation, redistribution of blood flow is achieved primarily by diameter changes due to contraction or relaxation of vascular smooth muscle in small arteries and arterioles, which result in large changes in resistance and flow according to Poiseuille's law (Secomb, 2008). Vascular tone is modulated by local concentrations of vasodilatory metabolites, autonomic influences, hemodynamic factors, and conducted responses from downstream vessels (Segal, 2005). Responses to hemodynamic factors include vasodilation in response to increasing wall shear stress (Pohl et al., 2000) as well as vasoconstriction in response to increases in transmural pressure (myogenic response) (Schubert and Mulvany, 1999).

Flow regulation during exercise additionally involves arteriolar dilation in active muscles due to metabolic factors, together with increased sympathetic stimulation causing vasoconstriction. The vasodilatory response of arterioles to hypoxia is thought to be due to the release of various metabolites from endothelium, tissue and erythrocytes in response to low oxygen levels (Davis et al., 2008). These local responses are augmented by communication of the generated signals along vessel walls, causing upstream vasodilation that increases oxygen delivery to hypoxic vascular beds (Segal and Jacobs, 2001; Secomb and Pries, 2002). Both upstream and downstream conduction of vasomotor signals have been demonstrated (Segal, 2000; Diep et al., 2005). Transmitted via gap junctions between endothelial and vascular smooth muscle cells (Gustafsson and Holstein-Rathlou, 1999), conducted responses are propagated over distances of order 1 mm or more (Bearden et al., 2004). Conducted responses have been shown to involve ATP sensitive potassium channels (Cohen and Sarelius, 2002) and can occur in the absence of erythrocytes (Ngo et al., 2010). Calcium-activated potassium channels have also been implicated (Behringer et al., 2013). Upstream conducted responses communicate the need for increased local blood flow to the vessels supplying a hypoperfused area and appear to be a critical component of flow regulation. Diffusive interactions between paired arterioles and venules may also play a role in coordinating arteriolar diameters with tissue needs (Hester and Hammer, 2002).

Sympathetic vasoconstriction is largely mediated via activation of α-adrenoreceptors on smooth muscle (Joyner and Casey, 2014). Adrenergic innervation of the arteriolar tree has been observed to extend to the level of terminal arterioles (Marshall and Hebert, 1986). During exercise, sympathetic vasoconstriction serves to maintain mean arterial pressure despite increased flow to active muscles (Laughlin et al., 2012). Attempts to increase oxygen delivery during exercise by pharmacologic vasodilation have resulted in either no improvement in maximal aerobic capacity (Barden et al., 2007) or decreased oxygen extraction (Lundby et al., 2008). Despite an increase in blood flow, vasodilation via adenosine results in impaired metabolism-perfusion matching (i.e., decreased extraction), likely due to shunting of blood to non-exercising tissue (Calbet et al., 2006; Barden et al., 2007). Similarly, experiments performed at altitude with adenosine vasodilation show a paradoxical worsening of oxygen uptake with a drop in extraction due to shunting (Lundby et al., 2008). These observations support the concept that sympathetic tone is instrumental in achieving metabolism-perfusion matching in exercise by maintaining blood flow to metabolically active areas and restricting blood flow to inactive areas (Calbet and Joyner, 2010). This implies that sympathetic vasoconstriction is overridden by other mechanisms in metabolically active tissues, a phenomenon known as “functional sympatholysis” (Remensnyder et al., 1962). One possible mechanism is the modulation of sympathetic vasoconstriction by vasodilatory metabolites (Marshall and Hebert, 1986; Joyner and Thomas, 2003; Thomas and Segal, 2004; Joyner and Casey, 2014), with the net effect of directing flow to metabolically active areas (Delp and Laughlin, 1998; Saltin et al., 1998; Sarelius and Pohl, 2010).

Experimental observations of the combined effects of sympathetic nerve activity (SNA) and muscle contraction were presented by VanTeeffelen and Segal (2003) in vessels of different caliber in hamster retractor muscle. At rest, sympathetic vasoconstriction resulted in a decrease in vessel diameter over approximately 2–3 s, reaching a steady state after about 20 s. The absolute diameter change was largest in feed arteries, while the relative diameter change was largest in third-order arterioles. The functional vasodilation that occurred with muscle contraction reached a steady state in approximately 1 min. It was found to increase from proximal to distal branches and to depend on the intensity of muscle contraction, indicated in terms of percent duty cycle (%DC) (VanTeeffelen and Segal, 2003). The effect of SNA on arteriole diameters was reduced during muscle contraction, demonstrating functional sympatholysis. In some instances, an initial constriction of the vessel during a period of SNA during muscle contraction was followed by a partial relaxation, an effect known as “sympathetic escape.” These effects depended on the location within the network. In feed arteries, vasoconstriction was relatively preserved, whereas a marked attenuation was seen at the level of third-order arterioles.

The mechanisms underlying this variation with vessel order are not definitely established. One hypothesis is that it results from variation of receptor subtypes. In some muscles, the sympathetic vasoconstriction of smaller vessels with higher concentrations of α_2_ adrenergic receptors appears to be more susceptible to inhibition by vasodilatory metabolites as compared to larger vessels with higher concentrations of α_1_ receptors (Anderson and Faber, 1991; VanTeeffelen and Segal, 2003). However, Rosenmeier et al. (2003) found that responses by both receptor subtypes were blunted in exercise. Furthermore, Moore et al. (2010) found that the distribution of receptor subtypes with vessel size is dependent on muscle type. These studies imply that variations with vessel size of receptor subtypes and their response to SNA may not be the primary reason for the dependence of sympatholysis on vessel size within the arteriolar tree.

The overall objective of the present study is to obtain improved quantitative understanding of flow regulation, including the effects of autonomic regulation. Toward this objective, a theoretical model for local blood flow regulation is developed that includes the multiple factors discussed above and their interactions. A simplified approach is used to describe network structure, in which vessels of different size ranges are represented by compartments in series, each compartment consisting of multiple identical vessels in parallel. This model allows simulation of the time-dependent responses of different orders of arterioles to SNA and functional vasodilation during exercise, and of the interactions between these stimuli, including functional sympatholysis and sympathetic escape.

The model presented here has potential applications for simulating and analyzing the regulation of blood flow under a range of physiological and pathological conditions in which autonomic regulation plays an important role, including exercise hyperemia and maldistribution of blood flow in sepsis. It provides a basis for modeling effects of autonomic regulation in heterogeneous network structures. In the present study, it is used to address the following hypotheses: (1) sympatholysis and sympathetic escape result from vasodilatory metabolic signals reaching the arterioles via conducted responses following sympathetic vasoconstriction; (2) the size-dependent variation of these phenomena results from variations in the strength of the metabolic signals reaching arterioles of different sizes via conducted responses.

## Materials and methods

### Model overview

**Figure 1:**
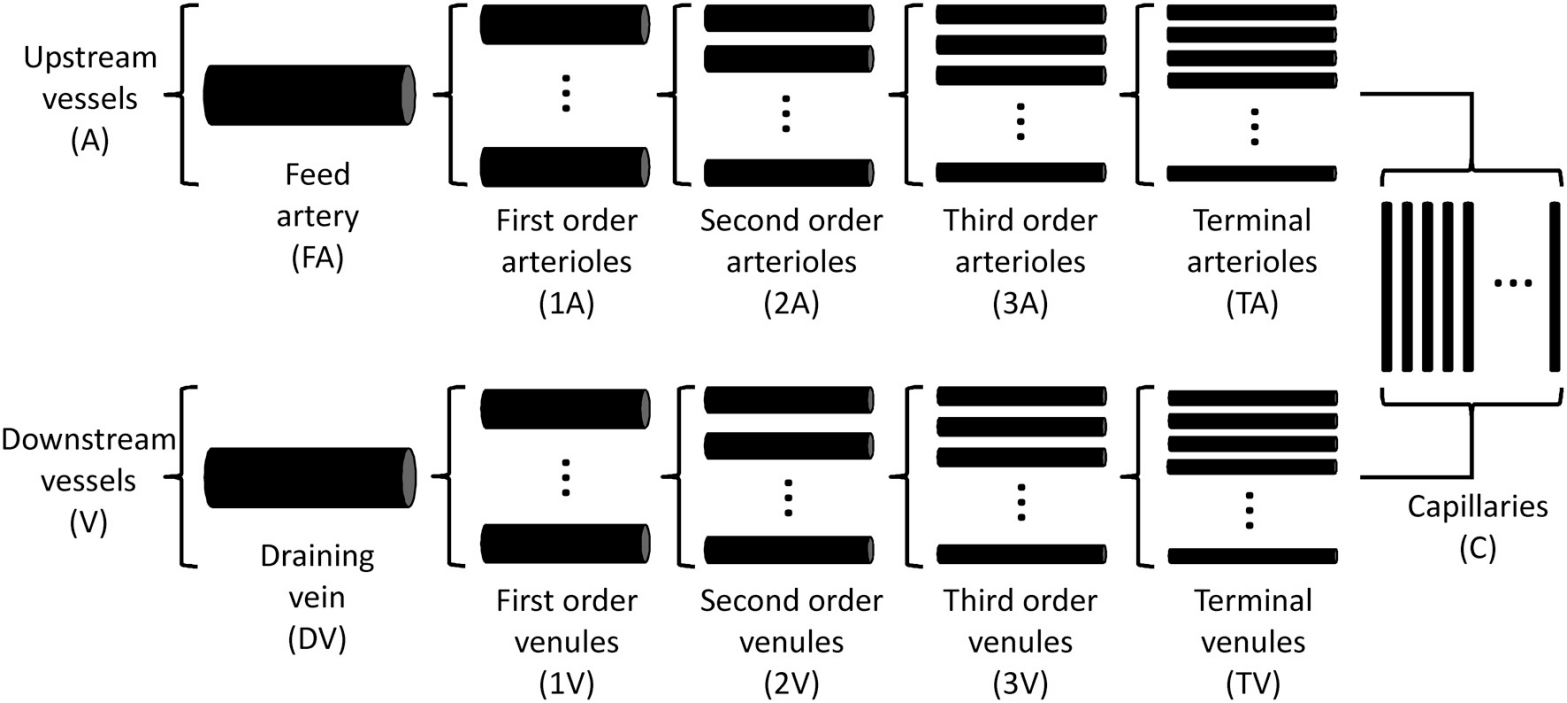
**Schematic diagram of representative segment model**. Each compartment is assumed to contain an array of identical segments in parallel, representing a range of arteriole or venule sizes.

A theoretical model was developed to investigate the interaction of sympathetic activity and functional vasodilation in the arteriolar tree. It is based on the “representative segment model” (Ursino et al., 1998; Cornelissen et al., 2002; Arciero et al., 2008; Carlson et al., 2008) in which vessels within different classes are assumed to have identical characteristics and responses. The seven-segment model (with two vasoactive segments) utilized in the studies of Arciero et al. (2008) and Carlson et al. (2008) was extended to include 13 segments, including five vasoactive arteriolar segments (Figure 1) in order to correspond to the arteriolar network of the hamster retractor muscle studied in VanTeeffelen and Segal (2003). Oxygen transport was assumed to occur in arteriolar and capillary segments according to the parameters in Table 1.

**Table 1:** **Values of model parameters related to oxygen transport and metabolic signaling**.

**Parameter**	**Description**	**Value**	**Units**	**References**
*M*_0_	Oxygen demand	1.0–8.28	cm^3^O_2_ cm^−3^ min^−1^	Arciero et al., 2008
*C*_0_	Oxygen carrying capacity	0.5	cm^3^O_2_ cm^−3^	McGuire and Secomb, 2001
*H*_*T*_	Tube hematocrit	0.3	–	Arciero et al., 2008
*H*_*D*_	Discharge hematocrit	0.4	–	Arciero et al., 2008
*R*_0_	Maximal ATP release rate	1.4 × 10^−9^	mol s^−1^ cm^−3^	Bergfeld and Forrester, 1992
*R*_1_	Effect of S on ATP release	0.891	–	Bergfeld and Forrester, 1992
*n*	Hill parameter	2.7	–	McGuire and Secomb, 2001
*P*_50_	Hill parameter	26	torr	McGuire and Secomb, 2001
*S*(0)	Initial oxygen saturation	0.97	–	Arciero et al., 2008
*C*_*ATP*_(0)	Initial ATP concentration	0.5	μM	Gonzalez-Alonso et al., 2002
*k*_*d*_	Rate of ATP degredation	2 × 10^−4^	cm s^−1^	Dull et al., 1992
τ_*d*_	Diameter time constant	1	s	Johnson, 1980
τ_*a*_	Activation time constant	20	s	Roy et al., 2012
*L*_*met*_	Conduction length constant	1	cm	Arciero et al., 2008
*C*^*meta*^_*rbc*_	Metabolic signal coefficient	30	μM^−1^ cm^−1^	Arciero et al., 2008

Parameters for each compartment (including vessel length, vessel number, and flow rate) were determined by specifying wall shear stresses and pressure drops in each segment (Table 2). The resultant pressure-diameter relationships are consistent with experimental observations (Pries et al., 1995).

**Table 2:** **Values of structural and hemodynamic parameters in the reference state for the representative segment model used in the simulations**.

**Parameter**	**A**	**FA**	**1A**	**2A**	**3A**	**TA**	**C**	**TV**	**1V**	**2V**	**3V**	**DV**	**V**	**Total**
Diameter, *D*, μm	100.7	**60**	**37**	**25.8**	**15.6**	**9.7**	**6**	14.93	24.63	43.19	65.21	109.4	183.7	
Wall shear stress, τ, dyn cm^−2^	**55**	**55**	**55**	**55**	**55**	**55**	**55**	**10**	**10**	**10**	**10**	**10**	**10**	
Pressure drop, ∆*P*, mmHg	**10**	**15**	**10**	**10**	**10**	**10**	**15**	1.18	1.15	1.09	1.03	1.50	1.00	86.94
Number of segments, *n*	0.23	**1**	4.33	14.62	93.06	585.3	4337	585.3	93.06	14.62	4.33	1	0.23	
Segment length, *L*, cm	0.61	0.5453	0.2242	0.1563	0.0945	0.0588	0.0545	0.0588	0.0945	0.1563	0.2242	0.5453	0.61	3.433
Velocity, *v*, cm s^−1^	3.053	1.976	1.201	0.7312	0.3141	0.1292	0.0456	0.0545	0.1260	0.2609	0.3866	0.5943	0.9174	
Viscosity, μ, cP	2.27	2.09	2.12	2.43	3.41	5.16	9.05	3.42	2.44	2.07	2.11	2.30	2.50	
Vascular volume, % of tissue	0.24	0.33	0.22	0.25	0.36	0.54	1.41	1.27	0.89	0.71	0.69	1.08	0.79	8.76

Values in bold are prescribed, and the remaining values are calculated based on geometric and hemodynamic considerations.

The model predicts diameter changes and oxygen delivery (perfusion) as a function of oxygen demand (consumption) and network perfusion pressure. Flow through the network is calculated assuming an overall pressure drop from the arteriolar inflow to the venular outflow. Arterioles are assumed to be vasoactive, whereas the capillary segments and venous vessels are assumed to act as fixed resistances (Arciero et al., 2008). Specific aspects of the model are discussed in the following sections.

### Flow regulation

The arterioles are assumed to regulate flow in response to metabolic, myogenic, shear-dependent and autonomic stimuli. Metabolic signals are assumed to be generated by release of ATP from erythrocytes at a rate that depends on oxyhemoglobin saturation (32, 37) and to be propagated upstream from all vessels to the arterioles via conducted responses. The specific assumed mechanism of metabolic response is not critical to the model, and other mechanisms providing sensitivity over a wide range oxygen levels would be expected to yield comparable results (Golub and Pittman, 2013). The diameter of each regulating segment is represented as a function of time by a model that incorporates vessel wall mechanics and vascular smooth muscle tone (Arciero et al., 2008; Carlson et al., 2008). This model is designed to represent the following key features of flow regulation by arterioles: (1) The vasoactive signal for tone generation depends on the balance between competing vasodilator and vasoconstrictor stimuli. (2) The level of vascular smooth muscle activation has maximal and minimal levels, and exhibits saturating behavior at high net levels of vasodilator or vasoconstrictor signals.

For each arteriolar segment, the generated wall tension *T*_total_ is assumed to consist of a passive component *T*_pass_ and an active component *T*_act_ in parallel such that

(1) $$T_{\text{total}}=T_{\text{pass}}+A\cdot T_{\text{act}}^{\text{max}}$$

where the activation *A* characterizes the level of vascular tone in each segment. For a vessel of diameter *D*, the passive component *T*_pass_ is given by

(2) $$T_{\text{pass}}=C_{\text{pass}}\exp\;[C_{\text{pass}}^{\prime }\left(D/D_{0}-1\right)]$$

where *C*_pass_ and *C*′_pass_ are constants and *D*_0_ represents the passive vessel diameter at a pressure *P* = 100 torr (Carlson and Secomb, 2005; Carlson et al., 2008). The active component *T*^max^_act_ generated by vascular smooth muscle is given by

(3) $$T_{\text{act}}^{\text{max}}=\pi r^{2}C_{\text{act}}\exp\;\left[-{\left(\frac{D/D_{0}-C_{\text{act}}^{\prime }}{C_{\text{act}}^{\prime \prime }}\right)}^{2}\right]$$

where *C*_act_, *C*′_act_, and *C*″_act_ are parameters characterizing the length dependence of force generation (Carlson and Secomb, 2005; Carlson et al., 2008). Values for the parameters describing vessel wall mechanics are given in Table 3.

**Table 3:** **Parameter values characterizing vascular smooth muscle properties for vasoactive segments**.

**Parameter**	**FA**	**1A**	**2A**	**3A**	**TA**
*D*_0_, μm	173.1	125.3	95.41	62.98	41.35
*C*_myo_, cm dyn^−1^	0.0079	0.0109	0.0143	0.0217	0.0331
*C*_shear_, cm^2^ dyn^−1^	0.0258	0.0258	0.0258	0.0258	0.0258
*C*_pass_, dyn cm^−1^	1154	834.9	636.0	419.8	275.6
*C*′_pass_	7.846	9.138	9.944	10.82	11.40
*C*_act_, dyn cm^−1^	2671	1654	1106	598.1	320.8
*C*′_act_	0.6709	0.6991	0.7167	0.7358	0.7486
*C*″_act_	0.2765	0.3148	0.3387	0.3646	0.3819

Numerical values of the parameters above are derived from formulas in Carlson and Secomb (2005) and Carlson et al. (2008); see also Table 1 of Fry et al. (2013).

The target activation *A*_total_ is calculated from

(4) $$A_{\text{total}}=\frac{1}{1+\exp\left(-S_{\text{tone}}\right)}$$

and satisfies 0 ≤ *A*_total_ ≤ 1, where *A*_total_ = 0 represents absence of vascular tone and *A*_total_ = 1 represents maximal vasoconstriction. The total vasoactive signal *S*_tone_ represents the net effect of the myogenic, shear-dependent, autonomic and metabolic responses on vascular smooth muscle tone:

(5) $$S_{\text{tone}}=C_{\text{myo}}T-C_{\text{shear}}\tau_{\text{wall}}+C_{\text{symp}}-S_{CR}+C_{\text{tone}}^{\prime \prime }$$

where *T* = *PD*/2 is the circumferential wall tension, τ_wall_ is the wall shear stress, *S*_CR_ represents the conducted (metabolic) response signal and *C*_myo_, *C*_shear_, *C*_symp_, and *C*″_tone_ are constants (Carlson and Secomb, 2005; Arciero et al., 2008). The time-dependent behavior of the diameter *D* and activation *A* in each segment are calculated by integrating the system of equations

(6) $$\frac{dD}{dt}=\frac{1}{\tau_{d}}\frac{D_{c}}{T_{c}}\left(T-T_{\text{total}}\right)$$

(7) $$\frac{dA}{dt}=\frac{1}{\tau_{a}}\left(A_{\text{total}}-A\right)$$

where *T* = *PD*/2, τ_*d*_ and τ_*a*_ are the respective time constants for changes in *D* and *A* and the subscript *c* refers to the control state (Carlson et al., 2008). In some cases, these solutions approach steady-state behavior, but oscillatory behavior is also possible (Arciero and Secomb, 2012).

### Oxygen transport

Oxygen transport in the network is calculated using a simplified model (Arciero et al., 2008). Zero-order oxygen uptake kinetics are assumed for a tissue sleeve of width 18.8 μm surrounding the feed artery and each arteriole and capillary, resulting in a linear decrease in oxygen saturation with distance until oxygen is fully depleted. The width of the sleeve corresponds to an overall capillary density of 500 mm^−2^, typical of skeletal muscle. The Hill equation is used to estimate oxygen saturation as a function of oxygen tension. Oxygen exchange is neglected in venous segments. Values for parameters governing oxygen transport are given in Table 1.

### Conducted metabolic response

Metabolic signals are assumed to be generated in all segments in response to the saturation-dependent release of ATP by erythrocytes (Ellsworth et al., 2009). Signals are assumed to be conducted upstream from all segments, including venules and capillaries, to the arterioles (Collins et al., 1998), where they influence the generation of vascular tone (Ellsworth et al., 2009). The local metabolic signal *S*_loc_ for each segment is:

(8) $$S_{loc}=C_{rbc}^{meta}\cdot C_{ATP}$$

where *C*_*ATP*_ is the mean local ATP concentration and *C*^*meta*^_*rbc*_ is a parameter describing the strength of the responses (see Table 1). The conducted signal *S*_*CR*_ (Equation 5) is assumed to act analogously to an electrical potential within the vessel wall, and is propagated upstream from the segment endpoint *x*_*end*_ with exponential decay according to a length constant *L*_*met*_ (Table 1), such that the conducted response *S*_*CR*_ at a given point *x* along the flow pathway in each segment is given by

(9) $$S_{CR}=\int_{x}^{x_{end}}e^{-(y-x)/L_{met}}S_{loc}(y)dy$$

and is continuous at the transitions from each class of vessels to the next. This method is appropriate for simulating conducted responses in representative segment models (Arciero et al., 2008), where all segments in a given compartment are equivalent. For heterogeneous networks, a more complex approach using circumference-weighted currents can be used (Roy et al., 2012). In some simulations, the values of the metabolic signals *S*_*CR*_ in each segment during SNA were artificially “locked” at the values that existed immediately prior to SNA. Comparisons with these simulations provided a basis for assessing the role of metabolic signals in sympatholysis.

### Autonomic regulation

The baseline diameter of arterioles is determined by the amount of muscle SNA at rest (Charkoudian et al., 2006). The effect of SNA increases above baseline on arteriolar tone is incorporated via the constant *C*_symp_ in Equation 5. Values of *C*_symp_ (1, 2, 4) were chosen corresponding to low, intermediate, and high (L, I, H) levels of SNA as generated by mean frequencies of 3, 6, and 12 Hz in the experiments of VanTeeffelen and Segal (2003). In simulations under resting conditions, the predicted decrease in diameter of first order arterioles (1A) with L, I, and H levels of SNA ranged from 3 to 10 μm. This is comparable to the reductions of 4–14 μm observed experimentally (VanTeeffelen and Segal, 2003), suggesting that the values of *C*_symp_ assumed in the model are appropriate.

### Reference state

Unlike the model of Arciero et al. (2008) and Carlson et al. (2008) which utilized a control state corresponding to moderate oxygen consumption, the reference state for the 13-segment representative segment model was chosen to correspond to resting conditions (Table 2). Resting diameters for arteriolar segments from feed arteries to third-order arterioles were specified based on observations of VanTeeffelen and Segal (2003). The diameter of the terminal arteriole was calculated as the geometric mean of the third-order arteriole and capillary diameters. Passive diameters were inferred by using interpolated ratios of resting to passive diameter for the vasoactive arteriolar segments in the model of Arciero et al. (2008). The values for *C*″_tone_ (Equation 5) used for each vasoactive compartment were determined in the reference state by setting the target activation *A*_total_ = *A* as deduced from Equation 1 and calculating *S*_tone_ from Equation 4.

### Oxygen demand

The metabolic vasodilatory stimulus resulting from muscle contraction was assumed in the model to act via increased oxygen demand and resulting reductions in intravascular oxygen levels. Oxygen demand values of 1, 1.91, 4.64, and 8.28 cm^3^O_2_ cm^−3^ min^−1^ were used to correspond to rest and to 2.5, 10, and 20% DC (contractile activity). The resulting steady-state diameters for vessels of different classes at different levels of consumption corresponded approximately to the experimentally observed values of VanTeeffelen and Segal (2003). Other mechanisms, such as release of potassium ions from contracting muscle, may also contribute to the metabolic response and could be included in this modeling approach (Lo et al., 2004).

## Results

**Figure 2:**
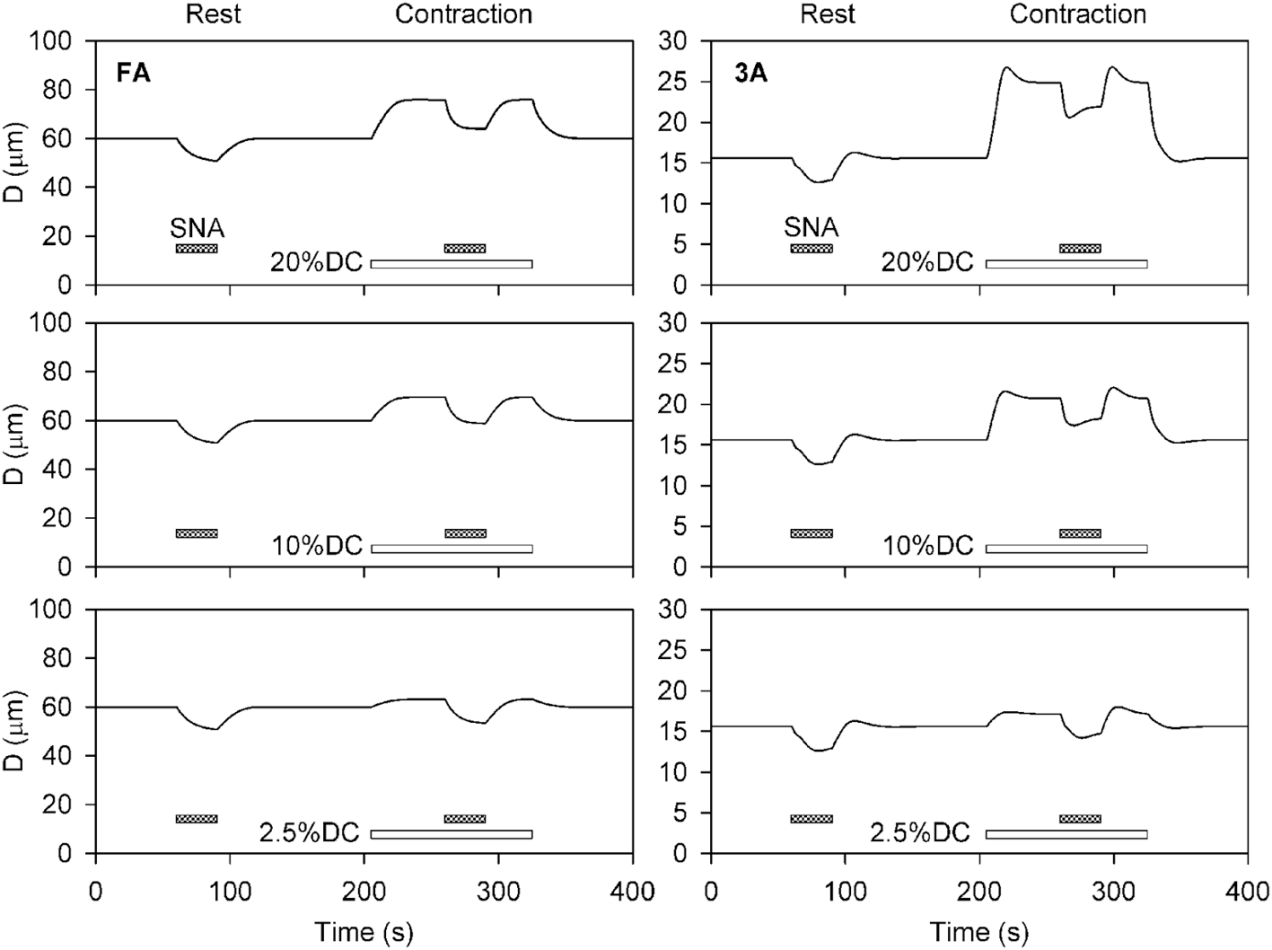
**Time-dependent diameter changes in feed arteries (FA) and third order arterioles (3A) in response to sympathetic nerve activity (shaded bars) at rest and with increased oxygen demand representing three levels of muscle contraction (unshaded bars)**. In all cases, results for intermediate levels of SNA (*C*_symp_ = 2) are shown. Assumed levels of oxygen demand were 1 at rest, and 1.91, 4.64, and 8.28 (all in units of cm^3^O_2_ cm^−3^ min^−1^) for the three levels of contraction (DC2.5, DC10, and DC20%).

Time-dependent simulations were performed for levels of SNA and contractile activity corresponding to the conditions in the experiments of VanTeeffelen and Segal (2003). Three levels of SNA and three levels of oxygen consumption were applied separately and in combination to investigate the interaction between SNA and contractile activity. Figure 2 shows the predicted time course of the diameters of the FA and 3A segments, under conditions corresponding to the experimental results shown in Figure 3 of VanTeeffelen and Segal (2003). In each case, a 30-s period of intermediate-level SNA is applied under resting conditions and during a period of increased oxygen consumption corresponding to muscle contraction. As expected, the model predicts vasoconstriction in response to SNA and vasodilation in response to increased oxygen demand in all cases. In the 3A segment, sympathetic escape is predicted both at rest and with each level of muscle contraction, as indicated by an initial vasoconstriction during SNA followed by a partial dilation. The model simulations show several features in common with experimental results (VanTeeffelen and Segal, 2003), including the prominent appearance of sympathetic escape in the 3A but not in the FA segment, and the occurrence of an after-dilation following the cessation of SNA. In the experiments (VanTeeffelen and Segal, 2003), one instance was seen of complete absence of vasoconstriction in response to SNA (in the 3A segment under contraction at 20% DC). According to the present model, such an effect would be possible due to a precise balance of opposing effects of SNA and metabolic dilation in a specific segment, but this did not occur with assumed parameter values.

**Figure 3:**
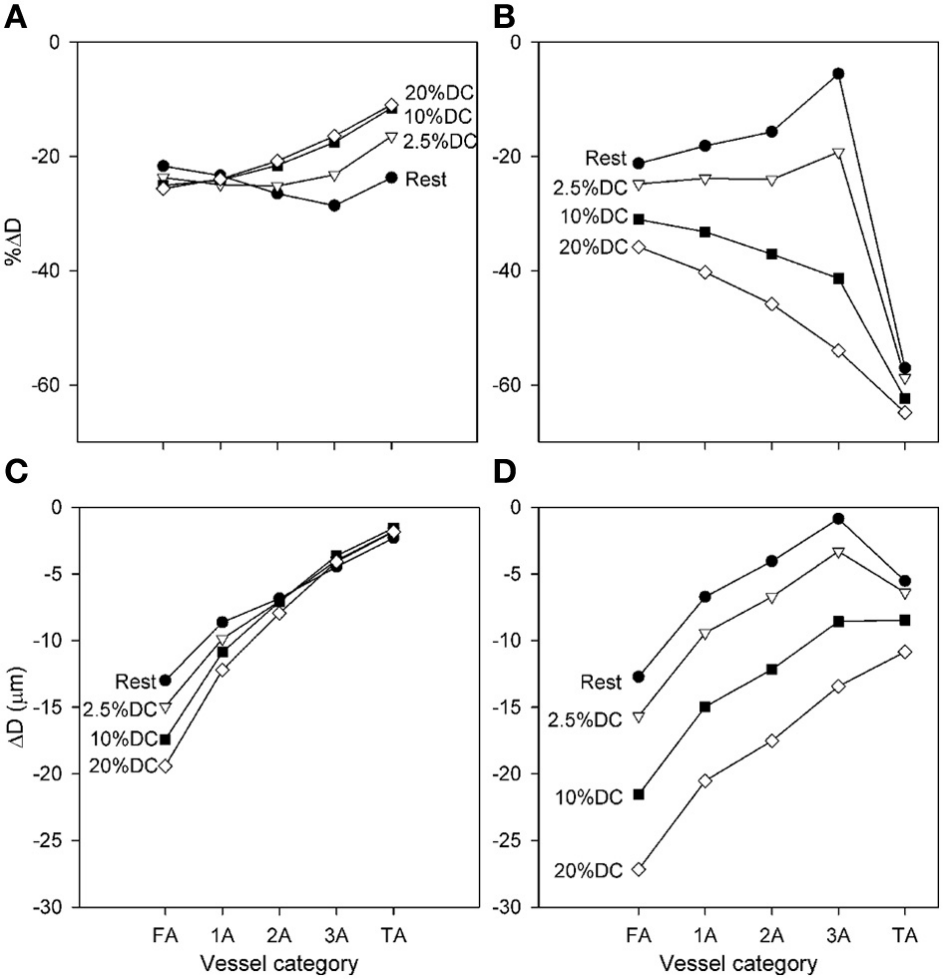
**Diameter changes with application of high SNA (*C*_symp_ = 4), as a function of arteriolar generation for different values of oxygen consumption. (A,C)** Results from model including normal metabolic responses. **(B,D)** Results from model with metabolic signals during SNA fixed at levels prior to application of SNA. In each case, relative **(A,B)** and absolute **(C,D)** Diameter changes are shown. All changes shown reflect the diameter at the end of SNA relative to the diameter established with increased oxygen consumption.

Predicted diameter changes of each arteriolar segment with application of high SNA (*C*_symp_ = 4) are shown in Figure 3 for several levels of oxygen consumption. Changes are shown for diameters at the end of SNA relative to the diameters prior to the period of SNA. Since these baseline diameters increased with increasing oxygen demand, the results are presented in terms of both percent and absolute reductions. The predicted diameter reductions with high SNA are in the range of 10–30% in all cases. The smallest percentage changes are seen in the smaller arterioles at higher levels of oxygen consumption, consistent with the more prominent effect of sympatholysis under those conditions. In absolute terms, the reductions in diameter varied from about 2–20%, with larger reductions in the larger arterioles. Figure 3 also shows predicted results when the metabolic signal is “locked” at its value before SNA, eliminating any compensatory metabolic signal generated in response to sympathetic vasoconstriction. In that case, the percentage reductions in diameter are generally larger, as much as 60% in the TA, and show a strong increase with muscle activation. Comparison of these sets of results implies that the variation of the conducted metabolic signal in response to reduction in flow plays a major part in moderating the contraction resulting from SNA.

**Figure 4:**
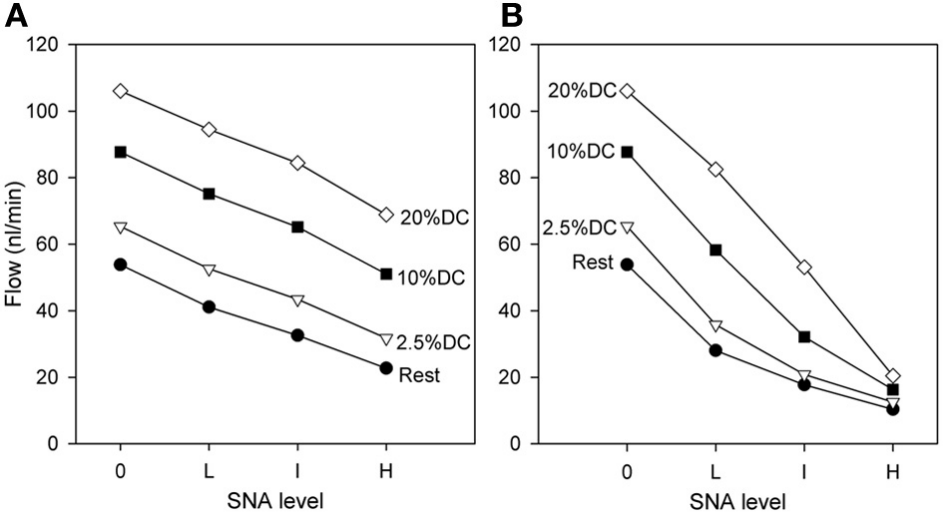
**Dependence of flow rate (at the end of SNA) through the network on oxygen demand and on level of SNA. (A)** Results from model including normal metabolic responses. **(B)** Results from model with metabolic signals during SNA fixed at levels prior to application of SNA. All changes shown reflect flows at the end of SNA relative to the flows established with increased oxygen consumption.

The predicted effect of SNA on blood flow rate through the network is shown in Figure 4. As expected, the flow rate at the end of SNA increases with the level of contractile activity, and decreases with increasing SNA. Moderate decreases in flow with SNA are seen when normal metabolic responses are included (Figure 4A). If, however, the metabolic responses are fixed at their respective levels prior to the application of SNA, the effect of compensatory metabolic vasodilation can be demonstrated. Under such conditions (Figure 4B), extreme decreases of flow are seen, particularly at higher oxygen demand. These results show the role of metabolic signals in maintaining flow during functional sympatholysis.

**Figure 5:**
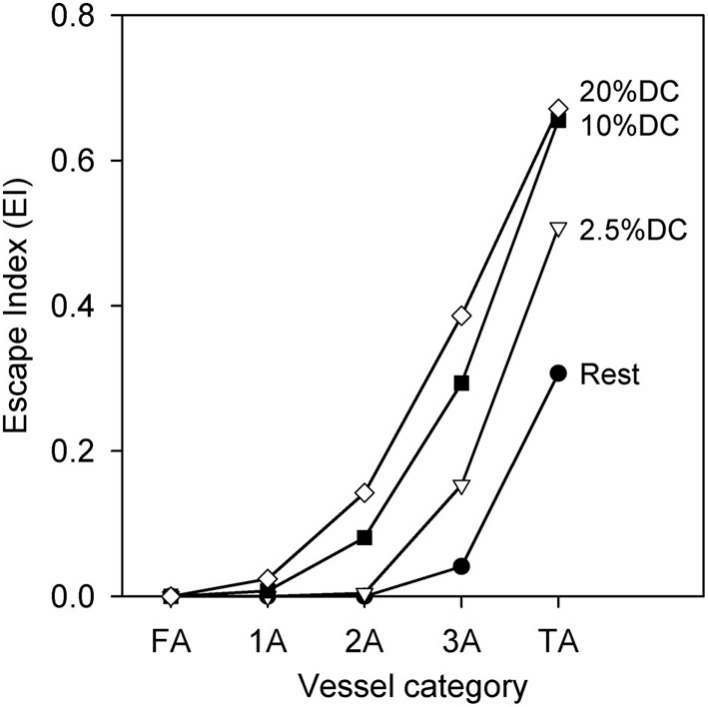
**Escape index (EI) [see text] as a function of arteriolar generation for different values of oxygen consumption at high SNA, demonstrating sympathetic escape**.

The phenomenon of sympathetic escape can be quantified using an escape index (EI) (Boegehold and Johnson, 1988), given by

(10) $$\text{EI}=\frac{D_{m}-D_{e}}{D_{m}-D_{0}}$$

where *D*_0_ represents the diameter prior to SNA, *D*_*m*_ represents the minimum diameter during SNA, and *D*_*e*_ represents the diameter at the end of SNA. Figure 5 shows that EI is larger for smaller vessels as compared to larger vessels, and that EI increases with increasing oxygen demand.

**Figure 6:**
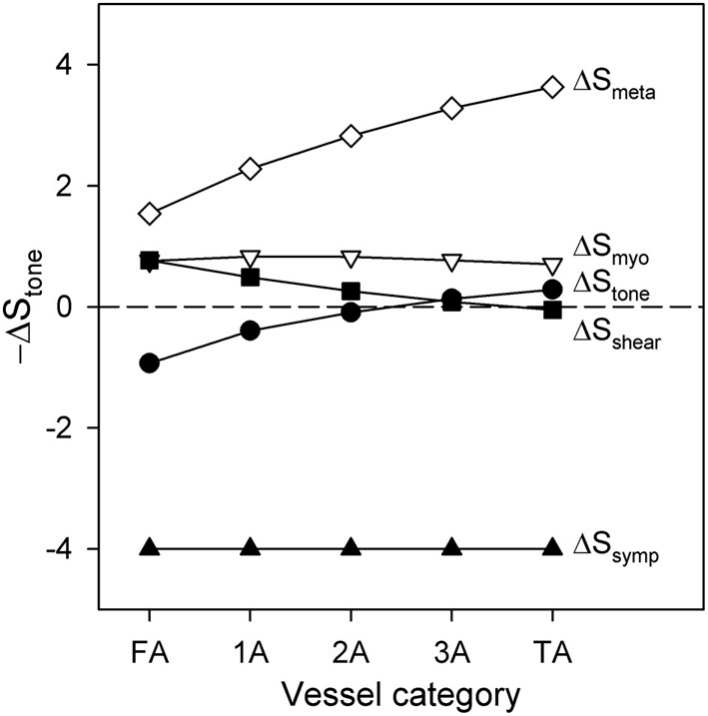
**Changes in the components of −∆*S*_tone_ and in total −∆*S*_tone_ following application of high SNA during a period of high contractile activity (20% DC), as a function of arteriolar generation**. Positive values of −∆*S*_tone_ indicate vasodilation, whereas negative values indicate vasoconstriction.

The contributions to functional sympatholysis of the various factors determining stimuli contributing to vessel tone are examined in Figure 6. Contractile activity corresponding to 20%DC is assumed. According to Equation 5, *S*_tone_ is the sum of the components *S*_myo_ = *C*_myo_*T*, *S*_shear_ = −*C*_shear_τ_wall_, *S*_symp_ = *C*_symp_, and *S*_meta_ = −*S*_CR_, along with an additive constant. Figure 6 shows the changes in each of these components in terms of the difference between the value at the end of 30 s of high-level SNA and the value prior to SNA. The changes in *S*_tone_ are displayed in terms of −∆*S*_tone_, such that higher values of this quantity represent vasodilation and lower values represent vasoconstriction. As Figure 6 shows, the vasoconstrictor effects of the SNA component *S*_symp_ are partially compensated by the metabolic vasodilator effect of *S*_meta_. This effect is strongest in the smaller arterioles, where *S*_tone_ is restored close to its pre-SNA level. As the metabolic signal is conducted upstream along the vessel walls, it gradually loses strength due to its distance-based decay (Equation 9). In the larger vessels, this results in a net vasoconstriction with SNA, i.e., −∆*S*_tone_ < 0. According to the model, this decay may be the cause of the reduced sympatholysis observed in the proximal arteriolar segments.

## Discussion

In exercising skeletal muscle, multiple mechanisms interact to increase perfusion, including increased cardiac output and peripheral vasodilation mediated by a range of metabolites. Sympathetic stimulation during exercise causes vasoconstriction of arterioles to actively contracting muscles, and plays a critical role in perfusion-metabolism matching at the microcirculatory level (Joyner and Thomas, 2003; Calbet and Joyner, 2010). Functional sympatholysis has the effect of directing blood flow to metabolically active areas. Impaired functional sympatholysis (as seen in aging, immobilization, and pathological states such as hypertension) has been implicated as a cause of poor metabolism-perfusion matching in skeletal muscle, resulting in performance limitations (Saltin and Mortensen, 2012), with the implication that this can be rectified by training (Jendzjowsky and Delorey, 2013). Some of the changes associated with aging may be due to decreases in the length constant for conducted metabolic responses, limiting the extent of vasodilation (Behringer et al., 2013). Although the physiological importance of functional sympatholysis is well accepted, its mechanistic basis and its role in exercise hyperemia remain the subjects of debate (Pancheva et al., 2013; Joyner and Casey, 2014). One obstacle is the difficulty of isolating the effects of interacting regulatory mechanisms in experimental systems.

In this context, theoretical models offer a potentially useful approach, providing a quantitative framework for integrating information about relevant mechanisms and examining their interactions. However, few previous theoretical studies have addressed the issue of autonomic regulation in the microcirculation. The present work uses a simplified model that nonetheless includes a number of important aspects of flow regulation in the microcirculation: (1) multiple mechanisms of flow regulation, including SNA; (2) a complete flow pathway, including arterioles of various sizes; (3) time-dependent behavior. The model approach builds on that developed previously (Arciero et al., 2008; Carlson et al., 2008). An advantage of this approach is that it provides a natural framework (Equation 5) for incorporating additional regulatory mechanisms, in this case the effect of SNA.

The main finding of this study is that the phenomenon of sympatholysis can be explained in terms of the negative feedback induced by metabolic flow regulation. When SNA is applied the resulting vasoconstriction results in a reduction in blood flow and impaired tissue oxygenation, which generates an upstream conducted vasodilator response that substantially compensates for the sympathetic vasoconstriction. A further finding is that a differential response to SNA based on vessel diameter need not be invoked to explain the observed dependence of sympathetic escape on arteriole size. In the model, the same sympathetic stimulus was applied to vessels independent of size, but the larger conducted metabolic signal in smaller vessels was responsible for functional sympatholysis. This arises as a consequence of the decay in the magnitude of the conducted metabolic response signal as it travels upstream to arterioles of successively increasing size. The decay rate may depend on the assumed length constant of the conducted response. Here *L*_*met*_ = 1 cm was assumed (Arciero et al., 2008). The vasoconstriction due to SNA in feed arteries overcomes metabolic vasodilation at higher oxygen demand, resulting in persistent vasoconstriction throughout the period of SNA. In smaller arterioles under similar conditions, however, the initial vasoconstriction with SNA is followed by partial relaxation, consistent with the experimentally observed phenomenon of sympathetic escape. This implies that the relative magnitude of metabolic responses can account for observed characteristics of functional sympatholysis, including the different responses of large and small arterioles.

The development of a theoretical model for flow regulation and sympatholysis necessarily involves numerous simplifying assumptions that may not accurately reflect the actual system. While these assumptions do not negate the general conclusions stated above, they may limit the ability of the model to provide a quantitatively correct representation of the system. Metabolic vasodilation is assumed to result from an oxygen-dependent mechanism and responses to other metabolic stimuli are not explicitly represented. The representative segment model used herein does not take into account a number of effects that are present in heterogeneous vascular networks, specifically (1) variation in flow pathways; (2) phase separation in bifurcations and formation of plasma channels; (3) capillary recruitment; and (4) regional variation in oxygen content. Finally, the simplified oxygen transport model used here does not include diffusive interactions between neighboring vessels.

The present model assumes that the metabolic signal originates from saturation-dependent ATP release from erythrocytes. Previous work has shown that such a mechanism does not provide optimal metabolism-perfusion matching in heterogeneous networks (Roy et al., 2012; Fry et al., 2013), and has suggested that wall or tissue-derived signals are important in metabolic flow regulation. Here, however, a homogeneous network structure is assumed, such that the results are not highly dependent on the assumed mechanism of metabolic regulation. The erythrocyte-derived signal was assumed because it has previously been shown to give results in good agreement with experimental data in a representative segment model (Arciero et al., 2008). The model could be readily modified to incorporate alternative mechanisms (Roy et al., 2012; Fry et al., 2013). A further assumption of the model is that the generation and upstream conduction of the metabolic signal are instantaneous. This may not be the case if metabolite generation and diffusion are responsible for the metabolic response. Signals with various time-dependent behaviors, such as adenosine (Mortensen et al., 2009), potassium (Lo et al., 2004), calcium (Behringer et al., 2013), and nitric oxide (Casey et al., 2013; Golub and Pittman, 2013), have been proposed as regulators of metabolic vasodilation under conditions of increased oxygen demand and may contribute to the phenomena of sympatholysis and escape. The time-dependent framework used in this model can readily be adapted to include the dynamics of metabolic signal generation to further investigate its interaction with SNA. The model provides a basis for investigating the interaction of metabolic and autonomic signals in pathological conditions such as sepsis, in which regional heterogeneity of oxygen demand, altered conduction of metabolic signals (Gustafsson and Holstein-Rathlou, 1999), and increased sympathetic activity are present.

### Conflict of interest statement

The authors declare that the research was conducted in the absence of any commercial or financial relationships that could be construed as a potential conflict of interest.
